# Laser Pyrolysis of Iron Oxide Nanoparticles and the Influence of Laser Power

**DOI:** 10.3390/molecules28217284

**Published:** 2023-10-26

**Authors:** Iulia Ioana Lungu, Ecaterina Andronescu, Florian Dumitrache, Lavinia Gavrila-Florescu, Ana Maria Banici, Iuliana Morjan, Anca Criveanu, Gabriel Prodan

**Affiliations:** 1 National Institute for Laser, Plasma and Radiation Physics, 409 Atomistilor Street, 077125 Magurele, Romania; iulia.lungu@inflpr.ro (I.I.L.); florian.dumitrache@inflpr.ro (F.D.); lavinia.gavrila@inflpr.ro (L.G.-F.); ana.niculescu@inflpr.ro (A.M.B.); iuliana.soare@inflpr.ro (I.M.); anca.criveanu@inflpr.ro (A.C.); 2 Faculty of Chemical Engineering and Biotechnologies, University Politehnica of Bucharest, 1-7 Gh. Polizu Street, 011061 Bucharest, Romania; 3 Academy of Romanian Scientists, Ilfov No. 3, 050044 Bucharest, Romania; 4 Electron Microscopy Laboratory, Department of Physics, Ovidius University of Constanta, 124 Mamaia Avenue, 900591 Constanta, Romania; gabiprodan@gmail.com

**Keywords:** laser pyrolysis, nanoparticles, experimental parameters

## Abstract

The purpose of this study was to investigate the synthesis of iron oxide nanoparticles under two different conditions, namely high and low gas flow rates, using laser pyrolysis and to examine the influence of laser power. The attained nanoparticles have been characterised regarding their stability and hydrodynamic dimensions by dispersive light scattering analysis (DLS), structure–X-ray diffraction (XRD), elemental composition–energy-dispersive X-ray spectroscopy (EDS) and X-ray photoelectron spectroscopy (XPS), and morpho-structural characterisation achieved by transmission electron microscopy (TEM) and selected-area electron diffraction (SAED). For a better understanding of the laser power influence, the residence time was also calculated.

## 1. Introduction

Nanoscience is essential for the research and development field in modern science. The various advantages provided by nanoparticles due to their size and features gives them a significant value. Magnetic nanoparticles, in particular, have gained considerable attention in fields such as medicine, biotechnology, engineering, and many more. Therefore, there is a constant search for appropriate production methods. Since their properties are strictly correlated with their size, the synthesis of uniformly sized nanoparticles is critical [1,2,3]. 

Iron oxide based nanostructures have been intensely studied due to their size-dependent physico-chemical properties. A critical factor that ultimately shapes the properties of the final nanoparticles (NPs) is the synthesis method [4,5]. Attaining high-purity and the controlled synthesis of iron oxide NPs is still a challenge. One of the most attractive characteristics of these nanoparticles is their magnetic behaviour; this behaviour depends on the physico-chemical properties of the resulting NPs, such as size, shape, chemical phases. The use of laser pyrolysis offers a promising alternative over conventional methods for the production of uniform NPs [6,7]. 

The efficiency and versatility of the CO_2_ laser pyrolysis of gaseous- or vapour-phase reactants regarding the synthesis of a wide selection of NPs, including iron oxides, has been greatly investigated for several applications. In order to attain iron oxide NPs using laser pyrolysis with the desired properties, iron pentacarbonyl (Fe(CO)_5_) is the most known precursor used [8,9,10]. Numerous studies have reported the use of iron oxide NPs synthesised with laser pyrolysis and their beneficial role in biomedical applications, amongst others [11,12].

Laser pyrolysis is mainly based on the resonance between the emission line of the CO_2_ laser and the IR absorption line of a gaseous/vapour-based precursor. If the precursor does not have an absorption band at the required wavelength, a separate agent, a sensitiser (generally, ethylene C_2_H_4_), can be used that has the role of an energy transfer agent. The role of the sensitiser is to absorb the energy from the laser and transfer it to the precursor through collision. The structure is based on a cross-flow configuration that can be found in the flow reactor. The reactants enter the above-mentioned configuration through a system of inner tubes. Finally, the reactant flow intersects with the laser beam, which is a continuously focused wave of CO_2_ laser radiation. There are several advantages of laser pyrolysis over conventional synthesis methods, including but not limited to: (i) the method being a one-step; (ii) the high purity of the final product; (iii) no sample preparation requirement when collecting; (iv) high yield and reproducibility; and (v) ease in controlling the experimental parameters. The versatility of producing nanostructures through this method is solely dependent on the choice of an appropriate precursor and the tuning of the experimental parameters. The precursors used are decisive in attaining the desired NPs; therefore, their selection is extremely important [10]. 

As mentioned earlier, iron oxide nanoparticles synthesised by laser pyrolysis have been intensely researched [8]. Due to the unique properties of these particles, and the versatility of this method, they have gained considerable attention, especially in the biomedical field [13]. There have been several reports on the properties and potential applications of laser-pyrolysis-synthesised iron oxide nanoparticles. Amongst the wide range of biomedical applications, cancer theranostics is one of the fields that could benefit from the unique properties of these nanoparticles. Superparamagnetic iron oxide nanoparticles have been intensely researched in cancer theranostics as imaging agents in MRI (Magnetic Resonance Imagining) and magnetic hyperthermia [14]. Bautista et al. studied the adsorption of dextran on the surface of iron oxide nanoparticles synthesised by both laser pyrolysis and co-precipitation. The results indicated that the dispersions revealed different results based on the synthesis method of the NPs. It was noted that a stronger bonding was formed on the surface of maghemite particles obtained from laser pyrolysis [15]. Morales et al. prepared iron oxide NPs by laser pyrolysis for their potential use as contrast agents. Using iron pentacarbonyl as a precursor and adjusting experimental conditions, they attained a narrow particle size distribution, with crystal sizes ranging from 2 to 7 nm [16].

Herein, we present the synthesis of iron oxide nanoparticle under two different conditions (high and low gas flow rates) using laser pyrolysis and the influence of laser power.

## 2. Results and Discussions

Previous parametric studies [17] showed that the temperature in the pyrolytic flame (mainly corresponding to the intersection area of the laminar gas flow and the focalised laser beam) strongly depends on the time spent by the gases in the reaction zone, which is also called residence time. Therefore, the study presented here maintains the same percentage ratio for the reactive gas flows (which enter the reaction chamber through the central nozzle) and evaluates the influence of power in two circumstances: low D_total_ for SFnew1-3: 70.8 sccm, and high D_total_ for SFnew4–5: 214.5. Moreover, in order to control the laminar flow of the central gases, a confinement Ar flow is calculated so that the speed at which they enter the reaction chamber is similar to the one of the reactive mix. 

Considering that the gas mixing process is an isothermal one, we have the following equation:

(1) $$D_{total}=\frac{D_{standard}\times p_{0}}{p_{work}}=\frac{\left(D_{C_{2}H_{4}}+D_{air}+D_{{Fe(CO)}_{5}}\right)\times p_{0}}{p_{work}}$$

However, $D_{total}=S\times v_{0}$, where

S—the area of the transversal section of the central admission nozzle (circular, with φ_int._= 0.19 cm);

*V*_0_—the initial speed of the gases when entering the reaction chamber.

Therefore,

(2) $$v_{0}=\frac{D_{standard}\times \frac{p_{0}}{p_{work}}}{\pi \times \frac{\varphi^{2}}{4}}\times \frac{0.1}{60}$$

In the reaction zone, the gases from the central flow dilate as a result of heating from the flame temperature T_fl_ (see Table 1). We consider this process an isobaric one because the gas pressure is very close with the one measured in the reaction chamber p_work_. Therefore, the movement speed of the laminar flow through the reaction zone is

(3) $$v_{fl}=\frac{v_{0}\times T_{fl}}{T_{0}}$$

Resulting in the final formula used to calculate the residence time, as follows:

(4) $$\tau =\frac{S\times \varphi_{fl}\times T_{0}\times p_{work}}{D_{standard}\times p_{0}\times T_{fl}}$$

By correlating the calculated residence time (Table 2) and the gas flow rates (Table 1), it is clear that a decrease in the total reactive flow leads to an increase in the residence time (SFnew4-D_total_ = 214.5 sccm, τ = 0.158 msec, while SFnew2-D_total_ = 70.8, τ = 0.559 msec). The laser power also affects the residence time, especially due to the increase in the reactive temperature with the increase in laser power. If we take the two circumstances separately (low and high D_total_), it can be observed that the higher the power, the lower the residence time (SFnew1–3: P = 150 W, τ = 0.449 msec; SFnew4–5: P = 86 W, τ = 0.158 msec). The increase in C at.% content can also be correlated with τ. For example, the highest residence time, corresponding to SFnew2, exhibits the most C at.% content for the first set of experiments, holding true for the second set as well (see Table 2 and Table 3). In the area of parametric variation presented here, the C content was minimal (see EDX analysis). It is usually present superficially, but in a sufficiently reduced quantity in order to not compromise the hydrophilic character of the as-synthesised NPs. Previous experiments at reaction temperatures over 700 °C lead to a significant C content that usually has a hydrophobic character, requiring an adequate stabiliser in order to attain a stabile suspension in aqueous media. Moreover, all the samples exhibit spectacular stability in water-based suspensions. Due to the low dimensions and large specific area, nanoparticles are prone to aggregate; this is especially encouraged by Brownian motion when in aqueous suspensions. Since DLS is mainly based on Brownian motion, and the fact that the results are for the hydrodynamic diameters and not the individual particles, this could be an explanation for the z-average values displayed in Table 4. 

Constant parameters throughout the experiments: pressure, p = 300 mbar; focal distance, d_fl_ = 28 cm; nozzle/flame diameters, φ_int_ = 1.9 mm, φ_ext_ = 3.00 mm, φ_fl_ = 2 mm; Ar gas flow, D_Ar conf._ = 750 sccm, D_Ar.window_ = 300 sccm for SFnew1–3, and D_Ar conf._ = 2000 sccm, D_Ar.window_ = 300 sccm for SFnew4–5. Abbreviations: P_Lar_, laser power in argon; P_Lab_, laser power in absorption; T_fl_, temperature of the pyrolytic flame.

**Figure 1:**
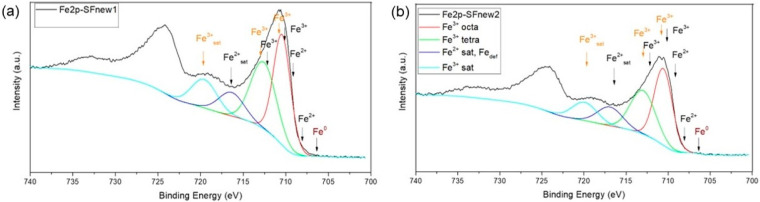
XPS spectra for iron: Fe2p-sensitive zone nanoparticles synthesised at (**a**) high laser power, SFnew1, (**b**) low laser power, SFnew2.

In Figure 1a,b, the high-resolution XPS spectra on the Fe2p-sensitive area for SFnew1 and SFnew2 powders are presented. The signal for the Fe2p 3/2 structure has been deconvoluted into four main peaks for Fe^3+^.

XPS analyses were performed in order to evaluate the differences in elemental composition at the superficial level (see Table 3), the mean elemental composition drawn by EDS spectra (see Table 5), and also samples synthesised under different conditions (laser power or total reactive flows). The main assumption is the high percentage of C (~30 at%) from XPS in comparison with its mean presence in whole particles. Taking into account that XPS analyses are mostly sensitive for elements and their bonding at the superficial level (~1 nm), we can conclude that the C atoms, which are most abundant on the surface, generate a hydrophobic character when the polymeric-like organisations prevail. Bomati-Miguel et al. published an interesting paper in 2008 that could lead to a correlation between the carbon and water content on the surface of maghemite nanoparticles. The nanoparticles in this work were synthesised by laser pyrolysis using iron pentacarbonyl as an iron precursor. The resulting particles were characterised by their reduced sizes (under 40 nm), with surface areas that reached up to 200 m^2^/g. Based on the obtained results, there is a clear correlation between the carbon content (which makes the surface hydrophobic) and the content of molar-absorbed water in two pairs of samples. The discussed pairs of samples have similar surface areas (~180 and ~200 m^2^/g), and the data shows that a higher carbon content leads to a lower water content. Therefore, confirming the assumption that the C atoms on the surface of the maghemite particles increases the hydrophobicity of the samples, whereas reducing it may lead to a higher water absorption, thus aiding their dispersion in water-based suspensions [18].

**Figure 2:**
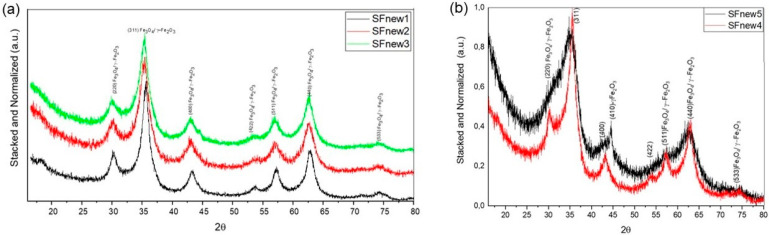
XRD patterns for (**a**) SFnew1–3 and (**b**) SFnew4–5.

A second assumption is the Fe/O ratio. In all cases, this value is lower than the value of Fe_2_O_3_, 0.66, which is the highest oxidate phase of iron. One relevant explanation might be the presence of functional groups, such as -OH, -C-OH -C=O, or -COOH. If such groups overcome the nanoparticle surface, they could facilitate the particle’s suspension in polar fluids (e.g., water) [19]. Moreover, they provide a preferable platform for further particle functionalisation with active biomolecules. Regarding the most probable iron ionisation state accordingly with the most probable crystalline phases, namely α-Fe^0^, γ-Fe^3+^_2_O^2−^_3_ or 2Fe^3+^Fe^2+^O^2−^_4_, HR (high-resolution) XPS analysis in the iron-sensitive area (700–740 eV) was performed (see Figure 2a (SFnew1-high laser power) and Figure 2b (low laser power)). Comparing the maximum position of Fe2p_3/2_ for maghemite and maghemite NPs, there is a shift of 0.5 eV between them, where the maghemite is placed at higher value, ~711.0 eV [20]. Here, we deconvolute in both spectra the larger peaks of Fe2p_3/2_ and Fe2p_1/2_ with four major peaks using the PseudoVoight fitting function. The resulting envelope curve is in good agreement with the experimental one. In Figure 1, only the fitting curves (the red, green, blue and cyan solid lines) for Fe2p_3/2_ were visualised. A comparison study between the maximum positions of the four resulting main peaks from the experimental spectra and those of the crystalline phases extracted from literature was performed [21,22,23]. For a clear view, we indicate the main peak corresponding to Fe 2p_3/2_ of Fe^0^ from α-Fe with brown, to Fe^3+^ and Fe^2+^ from Fe_3_O_4_ with black, and to Fe^3+^ from γ-Fe_2_O_3_ with orange. The absence of any contribution at less than 707 eV concludes that both samples have no reduced iron in its structure. The XPS spectrum for the Fe2p of the SFnew1 sample, between 707 and 709.5 eV, has to confer the contribution of Fe^2+^ ions from the octahedral sites, and the experimental data revealed that at least a minor contribution was present here. The Fe^3+^ contributions centred at 710.5 and 712.8 eV are placed between maghemite and magnetite peaks (octahedral and tetrahedral sites) but closer to those of maghemite. The contribution from the satellite zone (714–721 eV zone) is divided into two peaks: the major one is placed at 719.8 eV and comes mainly from Fe^3+^- maghemite ions, and the second one centered at 716.5 eV and might come from Fe^2+^ ions, from i.e., magnetite or lattice defects Fe_def_ [22]. In conclusion, the majority of the iron ion states at the NP surface level have to be maghemite in the case of the SFnew1 sample. Comparing the position-deconvoluted maxima of Fe2p_3/2_ for the SFnew2 sample (low laser power), the assumption that maghemite is the dominant phase became more evident. The main Fe^3+^ peaks (tetrahedral, octahedral and satellite) are placed very close with those of maghemite [22]. Furthermore, here we could not see any sign of contribution from Fe^2+^ ions at a binding energy lower than 709.5 eV.

Figure 2a shows the X-ray diffraction pattern of the laser-pyrolysis-synthesised nanoparticles. It exhibits a cubic structure, which may be indexed to the maghemite and magnetite phase Fe_3_O_4_/γ-Fe_2_O_3_ (JCPDS file 00-039-1346 and JCPDS file 00-019-0629). No other peaks for other oxides nor any traces of metallic phase (α-Fe) or carbides were noticed. 

The crystallographic structure of as-analysed NPs fits well with the magnetite/maghemite theoretical phases (PDF No: 00-019-0629 and 00-039-1346); both phases are of a spinel ferrite structure based on the cubic systems. The only differences derived from the lattice and cell volume constants are 8.396Å with 591.86 Å^3^ for magnetite and 8.352 Å with 582.50 Å^3^ for maghemite.

**Figure 3:**
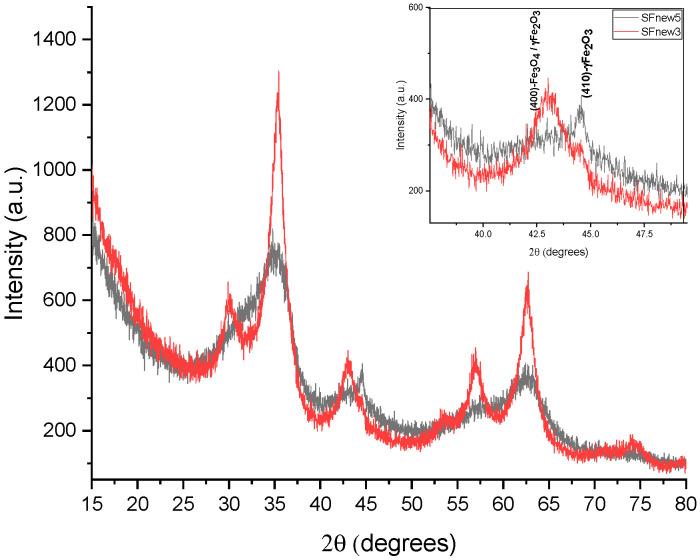
Close-up XRD patterns for SFnew3 and SFnew5.

Because the lattice parameters of magnetite and maghemite are extremely close, it is rather challenging to differentiate between them. The calculated lattice parameter for the attained samples, using the reflection of (311) and (440) peaks (see Table 6, column 3), generally display a value between both spinel structures. In both parametric studies, it is observed that the laser power favours the formation of the magnetite. Thus, for the sample SFnew2, which was synthesised at lowest laser power, the lattice constant is close to that of magnetite, a = 8.394 A, while the sample synthesised at a high power has a value close to that of a = 8.368 A. The SFnew3 and SFnew5 samples reveal the presence of a metallic iron phase by the peak at 44.49° (110) (Figure 3). 

The mean particle diameters were calculated from the XRD pattern according to the linewidth of the (311), (400) and (440) plane reflection peak using the Scherrer equation, as follows:

(5) $$D=\frac{K\lambda }{\;bcos\theta }$$

where *λ* is the X-ray wavelength (1.5418 Å), *b* is the full at half maximum of the XRD peak, and *K* is a shape factor, which is around 0.9 for magnetite/maghemite nanoparticles. The results are shown in Table 6, column 2.

The mean particle size can be correlated with the decrease in temperature of the pyrolytic flame induced by the laser power. The peaks on SFnew5 are noticeably broader, and the particles display dimensions of around 2 nm, with some reflections being difficult to distinguish. It can be observed that in both cases (Figure 3), the laser power strongly influences the crystallite dimension, with the dimension increasing with the increase in laser power, and clearly influences the reactive temperature.

**Figure 4:**
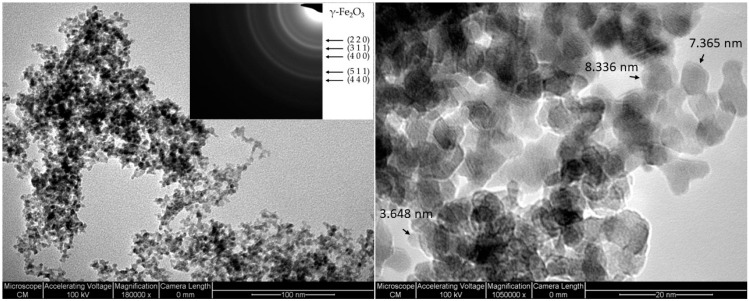
TEM micrographs of the synthesised NPs and SAED patterns in the insert.

The TEM micrograph for a reference sample (SFnew2) can be observed in Figure 4. The NPs exhibit a spherical morphology with an agglomeration tendency into chain-like aggregates, as it has been highlighted in previous papers as well [24]. ImageJ software 1.46r was employed for image processing, and a mean NP dimension of 4.2 nm was calculated. The bright ring pattern exhibited in the insert in Figure 4 indicates the nanocrystalline structure of the analysed sample. Moreover, the identified planes correspond with γ-Fe_2_O_3_ as was revealed with the XRD patterns. 

## 3. Experimental Part

### 3.1. Materials and Methods

#### Nanoparticle Synthesis

**Figure 5:**
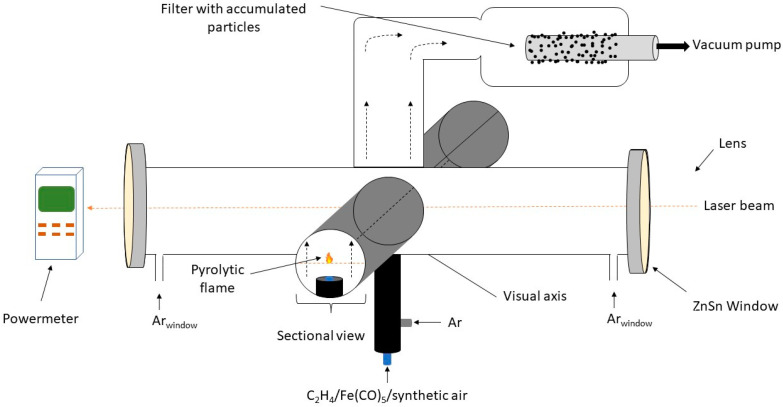
Experimental set-up of the laser pyrolysis installation for the synthesis of iron oxide nanoparticles [24].

The iron oxide nanoparticles were synthesised by laser pyrolysis, using the set-up presented in Figure 5. The iron precursor and oxidising agent used were iron pentacarbonyl (Fe(CO)_5_) (Merck, Darmstadt, Germany) and synthetic air, respectively. The sensitiser, or energy transfer agent, was ethylene (C_2_H_4_) (Linde plc., Munich, Germany). The method relies on the cross-flow configuration of the reaction chamber that enables the resonance between the emission line of a CO_2_ infrared laser and the absorption line of the gas sensitiser, therefore heating the reactive gases through collision energy transfer [10]. 

Concisely, the reactant flow orthogonally intersects the focused continuous wave of CO_2_ laser radiation (Coherent G Series with 400 W maximum output power, λ = 10.6 µm). A central inner tube delivers the mix of synthetic air and Fe(CO)_5_ vapours (brought by an ethylene flow). A coaxial argon (Ar) flow ensures the confinement of the gas precursors towards the flow axis, as well as the newly formed particles towards the collection chamber. The process gas has been detailed elsewhere [10]. Two sets of experiments were made: a series with a low total gas flow rate (SFnew1–3), and one with a high total gas flow rate (SFnew4–5). The experimental parameters are presented in Table 1. An optical pyrometer was used in order to measure the temperature in the pyrolytic flame. 

The gas flow for iron pentacarbonyl was calculated starting from the hypothesis that throughout the energetic process occurring during sparging, the carrier gas picks up a vapour flow with a percentual composition specific to the saturated vapours (Equation (6)). For Fe(CO)_5_, p_vap.sat._ is approximated at 38mbar and was calculated using the Antoine Equation for T = 25 °C = 298.15 K [25].

(6) $$D_{vap}=D_{carrier}\times \frac{p_{vap.sat.}}{\left(p_{work}-p_{vap.sat.}\right)}$$

### 3.2. Nanoparticle Characterisation

The hydrodynamic diameter and stability of the resulting nanoparticles in water-based suspensions have been analysed using dynamic light scattering (DLS) measurements (Nanoparticle Analyser SZ-100V2, Horiba, Kyoto, Japan). The apparatus operates a diode-pumped solid-state (DPSS) laser emitting at 532 nm, with a power of 100–240 V AC ± 10% at 50 Hz/60 Hz. The data are collected at a scattering angle of 173° due to the opaqueness of the obtained suspensions. The water-based suspensions were attained using the synthesised nanoparticles and distilled water at a concentration of 0.5 g/L, and placed in an ultrasound bath (59 kHz, 20 °C) with a cooling system operating for half an hour. All the measured suspensions reported a pH of around 5, while the blank distilled water had a pH = 6.79. The samples were measured in triplicate. 

X-ray diffraction (XRD) structural characterisation was attained using a Panalytical X’Pert Pro MPD, with a Brag–Bretano configuration. Continuous scanning was performed on an interval of (20°–80°) deg 2θ, with a 0.02° step size and an acquisition time of 20 sec/step at 45 kV and 40 mA. A divergent slit was used on the incident beam, and the use of a nickel filter and a curved graphite monochromator on the diffracted beam resulted in a monochromatic radiation CuKα of λ = 0.15418 nm. The mean crystallite dimension and the lattice parameter values were calculated using the Debye–Sherrer equation (ec. 5) using the HighScore Plus software Version 4.0 from the Panalytical and ICDD (International Centre of Diffraction Data) database.

The elemental analysis was attained using energy-dispersive X-ray spectroscopy (EDS) and X-ray photoelectron spectroscopy (XPS) analysis. For EDS, the NP powders were hand-pressed and fixed onto a copper foil. The analysis was obtained using an EDAX apparatus attached on a FEI Quanta Inspect S scanning electron microscope (SEM) at 10kV accelerating voltage.

XPS analysis was acquired using an ESCALAB Xi+ (Thermos Scientific Surface Analysis) equipment with an Al Kα radiation source (*h*ν = 1486.2 eV). The energy reference used was at the C 1s level (284.4 eV). The 5.978 version of the “Avantage” software was used to identify the superficial chemical compositions and oxidation states.

The morpho-structural characterisation was observed using transmission electron microscopy (TEM) and selected area electron diffraction (SAED) analysis employing a Philips CM 120ST (120 kV) apparatus.

## 4. Conclusions

This study investigated two sets of experiments, resulting in iron oxide nanoparticles and the influence of laser power on the final samples. The nanoparticles were synthesised by CO_2_ laser pyrolysis using iron pentacarbonyl as an iron precursor. In order to efficiently determine the influence of laser power on the synthesised nanoparticles, the total gas flow rates were varied. 

The structural characterisation of the attained nanoparticles has been investigated through XRD and XPS analysis, and it the Fe_3_O_4_/γ-Fe_2_O_3_ structure of the particles was confirmed. The residence time was calculated for each experiment, which resulted in a deeper insight on the effect of the experimental parameters on the final properties of the NPs. In the experimental conditions defined in this paper, nanoparticles with a low C content can be attained, with them being present mainly on the NPs surface. As a consequence of this, the NPs have a high tendency to disperse in water, obtaining agglomerate values under 200nm. It is worth mentioning that even though the nanoparticles might have aggregated in the water-based suspensions, it was not enough to deter their stability.

## Figures and Tables

**Table 1:** Experimental parameters.

Sample	Gas Flow Rates	Laser Power	Other Parameters
SFnew1	D_C2H4_ = 33 sccm D_air_ = 33 sccm D_Fe(CO)5_ ≅ 4.78 sccm	P_Lar_ = 157 W P_Lab_ = 150 W	T_fl_ = 670 °C
SFnew2	D_C2H4_ = 33 sccm D_air_ = 33 sccm D_Fe(CO)5_ ≅ 4.78 sccm	P_Lar_ = 80 W P_Lab_ = 75 W	T_fl_ = 485 °C
SFnew3	D_C2H4_ = 33 sccm D_air_ = 33 sccm D_Fe(CO)5_ ≅ 4.78 sccm	P_Lar_ = 125 W P_Lab_ = 120 W	T_fl_ = 680 °C
SFnew4	D_C2H4_ = 100 sccm D_air_ = 100 sccm D_Fe(CO)5_ ≅ 14.50 sccm	P_Lar_ = 88 W P_Lab_ = 86 W	T_fl_ = 608 °C
SFnew5	D_C2H4_ = 100 sccm D_air_ = 100 sccm D_Fe(CO)5_ ≅ 14.50 sccm	P_Lar_ = 40 W P_Lab_ = 35 W	T_fl_ = 400 °C

**Table 2:** Residence time calculated for the as-synthesised samples.

Sample	SFnew1	SFnew2	SFnew3	SFnew4	SFnew5
τ (msec)	0.449	0.559	0.445	0.158	0.208

**Table 3:** Superficial elemental composition evaluated with XPS analysis.

Sample	C (at.%)	O (at.%)	Fe (at.%)
SFnew1	28.8	50.3	20.9
SFnew2	30.1	50.2	19.7
SFnew3	28.0	47.1	24.9
SFnew4	28.8	46.1	25.1
SFnew5	30.0	46.3	23.7

**Table 4:** Mean hydrodynamic diameter (Z-average), polydispersity index (PDI), and NPs electrostatic repulsion (zeta potential) of the samples.

Sample	Z-Average (nm)		PDI		Z-Potential (mV)	
	Mean	SD	Mean	SD	Mean	SD
SFnew1	228.56	5.275	0.403	0.027	53.33	−0.543
SFnew2	181.5	6.481	0.465	0.063	61.43	0.418
SFnew3	135.6	1.070	0.359	0.009	61.9	0.989
SFnew4	141.6	1.608	0.416	0.051	58.93	1.172
SFnew5	162.26	5.441	0.398	0.006	60	0.355

Abbreviation: SD, standard deviation.

**Table 5:** Elemental composition obtained by EDS analysis.

**Sample**		**C (at.%)**	**O (at.%)**	**Fe (at.%)**
	**Element**			
SFnew1		0.63	53.2	46.17
SFnew4		0.91	42.72	56.37

**Table 6:** Structural data of the synthesised powders obtained from XRD analysis.

Sample	NPs Crystallographic Parameters		
	D_med_ (nm)	A (Å)	Vol_cell_
SFnew1	5.7	8.368	583.02
SFnew2	4.3	8.394	591.35
SFnew3	4.4	8.377	587.77
SFnew4	5.3	8.365	585.41
SFnew5	2.3	8.373	587.85

## Data Availability

No new data were created or analysed in this study. Data sharing is not applicable to this article.

## Sample Availability

Samples of the compounds are available from the authors.
